# Assessing the therapeutic potential and safety of traditional anti-obesity herbal blends in Palestine

**DOI:** 10.1038/s41598-024-52172-7

**Published:** 2024-01-22

**Authors:** Mohammed Hawash, Nidal Jaradat, Nihal Ayman Salhi, Beesan Shatreet, Areej Abu Asbah, Yousra Hijazi Hawash

**Affiliations:** 1 Department of Pharmacy, Faculty of Medicine and Health Sciences, An-Najah National University, Nablus, Palestine; 2 Lavender Care, Spa and Alternative Medicine Center, Nablus, Palestine

**Keywords:** Cancer, Drug discovery, Plant sciences, Diseases, Health care

## Abstract

The use of traditional herbal remedies has been a common practice for centuries across different cultures to treat various ailments. In Palestine, traditional herbal medicines are widely used, but their efficacy and safety have not been thoroughly investigated. Therefore, the purpose of this study was to assess the biological activity and toxicity of two traditional herbal blends often used to treat obesity in the West Bank region of Palestine. Two herbal blends with a total of eight plants were chosen based on their historic use and availability. The plant aqueous extracts were evaluated for their antioxidant, anti-fibrotic, anti-obesity, anti-diabetic, and cytotoxic activities. The results showed that these blends have potent antifibrotic, antioxidant, and anticancer activities. While their activities on α-amylase and lipase enzymes (main targets) showed moderate activities. Therefore, our results showed that Herbal Blend 2 was more potent than Herbal Blend 1 on all investigated targets. Herbal Blend 2 showed significant activities as an antioxidant, antifibrotic, and anticancer activities with IC_50_ values of 68.16 ± 2.45, 33.97 ± 1.14, and 52.53 ± 0.78 µg/mL against DPPH, LX-2, and MCF-7 cell lines, respectively. While it is IC_50_ values on α-amylase and lipase enzymes were 243.73 ± 1.57 and 1358.39 ± 2.04 µg/mL, respectively. However, the use of anti-cancer plants can be challenging due to their cytotoxic effects on the body. We urge individuals to exercise caution when using natural remedies and to seek medical advice before incorporating them into their health regimens. This study provides valuable insight into the potential health benefits of traditional herbal remedies and emphasizes the importance of responsible usage.

## Introduction

The West Bank of Palestine is home to a rich history of traditional herbal remedies used for centuries to treat various ailments. Despite their widespread use, these remedies have not been thoroughly evaluated for their safety and efficacy. Biological evaluation of traditional herbal remedies is essential to determine their potential as sources of novel therapeutic agents^1,2^. Traditional herbal remedies have been utilized for centuries to address various conditions such as obesity, diabetes mellitus, liver fibrosis, and cancer. They are believed to offer several advantages over modern medicine^3^.

Obesity is a major health concern globally, and the use of herbal supplements is gaining increasing popularity as a complementary therapy. Several herbal supplements, such as green tea, cinnamon, and turmeric have been found to have anti-obesity effects through various mechanisms, including reducing appetite, increasing energy expenditure, and enhancing lipid metabolism^4,5^.

Diabetes mellitus (DM) is a chronic metabolic condition affecting millions of people throughout the world^6,7^. The use of herbal supplements as an adjunct to conventional therapy has gained popularity due to their potential antidiabetic effects and fewer side effects. Some herbs, such as ginseng, fenugreek, and bitter melon have been found to have hypoglycemic effects through various mechanisms, including enhancing insulin secretion and sensitivity, and reducing glucose^8,9^.

Liver fibrosis is a progressive disease that can lead to liver cirrhosis and liver failure. Several chemicals and plant extracts have been shown to have anti-fibrotic effects on LX-2 cell lines. Some of these include; Curcumin, an extract from turmeric, which has been shown to have anti-fibrotic effects on LX-2 cells. Epigallocatechin gallate (EGCG), a compound found in green tea, has been shown to inhibit LX-2 cell proliferation and fibrosis. Quercetin, a natural flavonoid found in fruits and vegetables, has been shown to have antifibrotic effects on LX-2 cells by reducing oxidative stress and inflammation. Resveratrol, a compound found in red wine and grapes, has been shown to inhibit LX-2 cell proliferation and fibrosis^10,11^.

Oxidative stress is a major contributor to the development of several chronic diseases. Herbal supplements have been found to have potent antioxidant effects that can reduce oxidative stress and prevent the development of various diseases. Some herbs such as turmeric, ginger, and garlic have been found to have potent antioxidant effects through various mechanisms such as scavenging free radicals, enhancing endogenous antioxidant enzymes, and reducing oxidative damage to cellular components^12,13^.

Cancer stands as a significant contributor to both illness and death in Palestine, where the predominant type among males is lung cancer, and among females is breast cancer. The complexity of cancer manifests through diverse underlying mechanisms^14^. The use of herbal remedies as complementary therapy for cancer has gained popularity due to their potential anticancer effects. Several herbs such as green tea, ginger, and garlic have been found to have potential anticancer effects through various mechanisms such as inducing apoptosis, inhibiting angiogenesis, and reducing oxidative stress^15,16^.

The eight different medicinal plants include *Camellia sinensis* L. Kuntze, *Zingiber officinale* Roscoe*, Cuminum cyminum* L, *Anthemis cotula* L., *Cinnamomum verum* J. Presl, Natural Apple (*Malus sylvestris* Mill.) cider vinegar*, Curcuma longa* L, *Allium sativum* L., and *Piper nigrum* L. have biological activities on various biological targets as listed in Table 1.

**Table 1:** The plants’ scientific names, common names, and biological activities.

Scientific Name and voucher specimen code	Common name	Biological activities
*Cinnamomum verum* J. Presl [Pharm-PCT-2707]	Cinnamon	It has anticancer, antioxidant, antilipid, and antifibrotic effects^17^. It has been found to have anti-cancer effects against various types of cancer cells, including breast cancer by inducing apoptosis and suppressing angiogenesis, lung, and colon cancer cells^18,19^
*Curcuma longa* L. [Pharm-PCT-27092707]	Turmeric	It possesses powerful anticancer properties and has been proven to suppress the growth of numerous cancer cells, including breast, prostate, and colon cancer cells. *C. longa* also contains high antioxidant activity, which aids in the prevention of oxidative stress-related disorders like cardiovascular disease and cancer. *C. longa* also has anti-lipid properties and has been demonstrated to lower blood glucose and lipid levels in diabetic mice. Finally, *C. longa* contains antifibrotic property that may be beneficial in the prevention and treatment of fibrotic illnesses such as liver fibrosis^20,21^
*Allium sativum* L. [Pharm-PCT-2704]	Garlic	Its bioactive components prevent the growth of cancer cells, such as breast, prostate, and colon cancer cells. The ORAC (Oxygen Radical Absorbance Capacity) experiment revealed that garlic extracts have excellent antioxidant activity. Garlic has antifibrotic actions and has been proven to lower blood glucose and cholesterol levels in diabetic rats. It also has anti-inflammatory activities and may be effective in the prevention and treatment of fibrotic disorders^22^
*Camellia sinensis* L. Kuntze [Pharm-PCT-2706]	Tea	It has an anticancer, antioxidant, antiipidemic and antifibrotic properties. Tea catechins induce apoptosis in cancer cells, decrease angiogenesis (the creation of new blood vessels that feed tumors), and modulate cell signaling pathways involved in cancer growth and progression. Green tea extract reduces serum triglycerides, total cholesterol, and LDL cholesterol levels in human subjects. Moreover, It inhibits the activation of hepatic stellate cells, which are responsible for the excessive accumulation of extracellular matrix proteins in the liver, and hence reduces liver fibrosis in rats^23–25^
*Zingiber officinale* Roscoe [Pharm-PCT-2724]	Ginger	It has anticancer, antioxidant, antilipidemic, and antifibrotic properties. Gingerols, the major bioactive compounds in ginger, inhibit the growth and proliferation of colon cancer cells by inducing apoptosis and cell cycle arrest. Moreover, ginger has been found to enhance the anti-cancer effects of chemotherapy and radiation therapy by reducing the side effects of these treatments^26–29^
*Cuminum cyminum* L. [Pharm-PCT-2776]	Cumin	It has anticancer, antioxidant, antilipidemic, and anti-ibrotic properties. Cumin extract has high antioxidant activity, as measured by the DPPH and ABTS assays, and can prevent lipid peroxidation and DNA damage induced by oxidative stress^30^
*Anthemis cotula* L. [Pharm-PCT-178]	Stinking chamomile	It is a medicinal plant that has been traditionally used for various ailments. It possesses several health benefits, including anticancer, antioxidant, antilipidemic and antifibrotic properties^31,32^
*Piper nigrum* L. [Pharm-PCT-2790]	Black pepper	It has anticancer activity due to the presence of piperine which can inhibit the growth of cancer cells, induce apoptosis, and prevent the formation of new blood vessels. It is effective against various types of cancer including breast cancer, prostate cancer, and colon cancer. Piperine has also been reported to increase the activity of antioxidant enzymes, such as superoxide dismutase (SOD) and catalase, which further enhance the antioxidant defense mechanism of the body. Moreover, white pepper has an anti-lipid and anti-fibrotic effect^33^
*Malus sylvestris* Mill	Natural Apple (cidar) vinegar	Vinegar contains acetic acid, which has been shown to have anticancer properties by inducing apoptosis, inhibiting angiogenesis, and suppressing tumor growth in vitro and in vivo studies. Moreover, vinegar has been found to possess antioxidant properties by reducing oxidative stress and scavenging free radicals. The antilipidemic effect of vinegar have also been demonstrated in various animal studies where it was shown to reduce blood lipid levels. Additionally, vinegar has been shown to possess antifibrotic effects by reducing collagen accumulation in liver fibrosis^34,35^

Our decision to formulate a polyherbal Blend stems from the longstanding traditional use of such blends in our local market for addressing obesity-related concerns. These traditional formulations often passed down through generations, have garnered popularity due to anecdotal evidence suggesting their efficacy in managing obesity. However, we recognize that relying solely on historical usage is not sufficient in the context of modern scientific research. As reported, herbal supplements have shown promising effects in the management of obesity, DM, liver fibrosis, oxidative stress, and cancer. Therefore, the current work aims to evaluate two common traditional herbal blends in the West Bank of Palestine, which are consumed by people for obesity, and also assess their effects on different biological targets.

## Material and methods

### Plant collection and extraction

The Herbal blends 1 and 2 were taken in November 2021 from a Palestinian apothecary in the West Bank. The plants were identified by a pharmacognosist Prof. Dr. Nidal Jaradat and voucher specimens were deposited at the Natural Products Laboratory of the Faculty of Medicine and Health Sciences at An-Najah National University and kept under the herbarium voucher specimen number: Pharm-PCT-178, Pharm-PCT-2704, Pharm-PCT-2706, Pharm-PCT 2707, Pharm-PCT-2709, Pharm-PCT-2724, Pharm-PCT-2776, Pharm-PCT2790 as mentioned before in Table 1. All methods were carried out in accordance with applicable institutional, national, and international guidelines and legislation. The plant portions were stored in the shade at a regulated humidity (55 ± 5 RH) and temperature (25 ± 2 °C). Each herbal Blend was mixed in an equivalent ratio of each plant and **Herbal Blend 1** included *C. sinensis, Z. officinale, C. cyminum, A. cotula, C. verum,* and *natural vinegar*. While **Herbal Blend 2** included *C. verum, C. longa, A. sativum,* and *P. nigrum.* All of these plants extracts showed biological activities on various biological targets as listed in Table 1**.**

### Instrumentation

A Spectrophotometer-UV/Visible (Jenway® 7135, Staffordshire, UK), filter papers (Whitman No. 1, Washington, USA), Shaker device (Memmert 531-25-1, Stockholm, Germany), rotavap apparatus (Heidolph-VV 2000, Schwabach, Germany), grinder (Aero Plus 500 W Mixer Grinder, I01, Wan Chai, China), electronic-balance (Radwag, AS 220/c/2, Toruńska, Poland) and Cryo-Desiccator (Mill-rock technology, BT85, Kingston, USA) were used.

### Chemicals

Loba Chemie (Mumbai, India) provided acetone, sodium hydroxide, n-hexane, and methanol, while Alfa Agar (Binfeld, UK) provided Ninhydrin solution, Benedict’s, and Millon’s reagents. Alfa Aesar (Lancaster, UK) also provided iodine solution, sulfuric acid, and Molisch’s reagent. Sigma-Aldrich (Steinheim, Germany) provided the Folin-Ciocalteu’s reagent, hydrochloric acid, aluminum chloride, potassium acetate, chloroform, and 2,2-diphenyl-1-picrylhydrazyl (DPPH). Riedel-De-Haen (Teningen, Germany) provided magnesium ribbon, acetic acid, ferric chloride, and dimethyl sulfoxide (DMSO). Trolox ((*s*)-(-)-6-hydroxy-2,5,7,8-tetramethychroman-2-carboxylic acid) and quercetin were also obtained from Sigma-Aldrich (Sborg, Denmark). α-amylase, on the other hand, was brought in from Sigma (Mumbai, India). Sigma (St. Louis, USA) provided the DNSA (3,5-dinitro salicylic acid) reagent, Acarbose, *p*-nitrophenyl butyrate, Orlistat, tris–HCl buffer, and Porcine pancreatic lipase type II.

### The water solvent fractionation method

Water is commonly used as a solvent in traditional herbal medicine due to its availability, non-toxicity, and ability to extract a wide range of compounds. It is also considered to be more representative of the traditional method of preparing herbal remedies^36^. The powdered substance of the two herbal blends was fractionated progressively by adding water (polar-protic solvents): Approximately 50 g of each Blend was steeped in 500 mL of water separately, and the fraction was shaken for 72 h at room temperature at 100 rounds/min. After that, it was refrigerated for 6 days. A Cryo-Desiccator was used to lyophilize the aqueous fraction. Finally, until future usage, all crude plant fractions were kept in the refrigerator at 4 °C.

### Antioxidant

Activity For the evaluation of Herbal Blend fractions and Trolox (positive control), a concentration of 1 mg/mL in methanol was first produced from the Herbal blends. The generated solution was used to make concentrations of 5, 7, 10, 20, 30, 50, 80, and 100 µg/mL. The DPPH (2,2-diphenyl-1-picrylhydrazyl) reagent was then diluted in 0.002% w/v methanol and combined with the previously prepared working concentrations in a 1:1:1 ratio. The pure methanol solution served as a control. All of the solutions were incubated in a dark environment at room temperature for 30 min. The absorbance readings were then calculated using a UV–visible spectrophotometer with a wavelength of 517 nm. The percentage of antioxidant potential of each plant fraction and Trolox was calculated using the following formula:

$${\text{DPPH radical percent of inhibition }} = \, \left[ {{\text{A}}_{{\text{C}}} - {\text{ A}}_{{\text{S}}} } \right]/{\text{ A}}_{{\text{C}}} *{1}00$$

A_C_ is the absorbance of the control; A_S_ is the absorbance of the tested samples. Using BioDataFit edition 1.02^18^, the antioxidant half-maximal inhibitory concentration (IC_50_) of each Herbal Blends was computed^37,38^.

### Porcine pancreatic lipase inhibition assay

The anti-lipase assay was carried out following the findings of^39^ with slight modifications. In brief, a stock solution was generated by combining 1 mg/mL of each Herbal blend’s component with 10% dimethyl sulfoxide, from which concentrations of 50, 70, 500, 700, 1000, and 1500 µg/mL were prepared. A pancreatic lipase stock solution of 1 mg/mL was also combined with a Tris–HCl buffer solution. 20.90 mg of *p*-nitrophenyl butyrate was suspended in 2 mL of acetonitrile to make a stock solution. Then, 0.2 mL of plant fraction was mixed with 0.1 mL of porcine pancreatic lipase enzyme (1 mg/mL). The resulting herbal blends were then diluted to 1 mL with a Tri-HCl solution and stored at 37 °C for 15 min. Following that, each working Herbal Blend received 0.1 mL of *p*-nitrophenyl butyrate. These herbal mixes were incubated at 37 °C for 30 min. The hydrolysis of *p*-nitrophenolate to *p*-nitrophenol at 405 nm was calculated using a UV/visible spectrophotometer to assess pancreatic lipase activity. Furthermore, all of the Herbal mixes investigated were tested in triplicate.

### α-amylase inhibitory assay

This process was conducted using a modified strategy. A 200 µL aliquot of each Herbal blends portion at 50, 70, 500, 700, 1000 and 1500 µg/mL concentrations was placed in a test tube with 200 µL of 0.02 M sodium phosphate buffer (pH 6.9) containing-amylase solution (2 units/mL). After 10 min at 25 °C, 200 µL of 1% starch solution mixed with 0.02 M sodium phosphate buffer solution (pH 6.9) was added at scheduled intervals and held for 10 min at 25 °C. The reaction was halted by the addition of 200 µl of DNSA.

These tubes were then placed in boiling water for 5 min before being cooled to room temperature. The herbal blends were then diluted with 5 mL of distilled water, and the absorbance at 540 nm was calculated using a UV–visible spectrophotometer. The same process was used to make a control herbal mix, but the plant fraction was replaced with distilled water. Using the following equation, the α-amylase inhibitory activity was determined as a percentage of inhibition: The amounts of plant components required to inhibit lipase enzyme activity by 50% (IC_50_) were calculated graphically. The same procedure was followed for the positive control of α-amylase inhibitory activity, Acarbose^40,41^.

### Cell culture and cytotoxicity assay

The liver (Hep3B), breast (MCF-7), and human cervical (HeLa) tumor cell lines were grown separately in RPMI-1640 media (Sigma, Norwich, UK), which was supplemented with 1% l-glutamine (Sigma, London, UK), 1% penicillin/streptomycin antibiotics (BI, New Delhi, India), and 10% fetal bovine serum. Cancer cells were cultured in a humidified environment with 5% CO_2_ at 37 °C. In a 96-well plate, cells were planted at 2.6 × 10^4^ cells/well. Cancer cells were cultured for 24 h with various concentrations (10, 50, 100, 250,100, 500, 2000, and 4000 µg/mL) of both herbal blends after 48 h. Cell viability was determined using the CellTilter 96® Aqueous One Solution Cell Proliferation (MTS) Assay (Promega Corporation, Madison, WI, USA) according to the manufacturer’s instructions. After the treatment, 20 µL of MTS solution per 100 µL of the medium was added to each well, and the well herbal blends were incubated at 37 °C for 2 h. At 490 nm, the absorbance was measured^42,43^.

### Statistical analysis

The antioxidant, anti-lipase, anti-α-amylase, cytotoxic, and anti-fibrotic activities of the eight plant fractions tested were all given as mean SD standard deviation; the result was judged significant when the *p*-value < 0.05, as well as the *p*-values were calculated by using *t*-test function accordingly.

## Result and discussion

The increasing use of traditional medicinal herbs has raised concerns about potential side effects and misapplication. Herbal products have been linked to a variety of negative side effects, including life-threatening conditions and serious injuries. Some of the difficulties in monitoring the safety of herbal remedies stem from the lack of mandatory safety evaluations before marketing, as well as the lack of quality standards regulations, and effective manufacturing practices in many countries^44,45^. Therefore, our study focused on the biological evaluation of two traditional herbal remedies containing eight herbal plants commonly used in traditional Palestinian medicine in the West Bank of Palestine and evaluate their anti-obesity, antidiabetic, antifibrotic, antioxidant, and anti-cancer effects.

**Figure 1:**
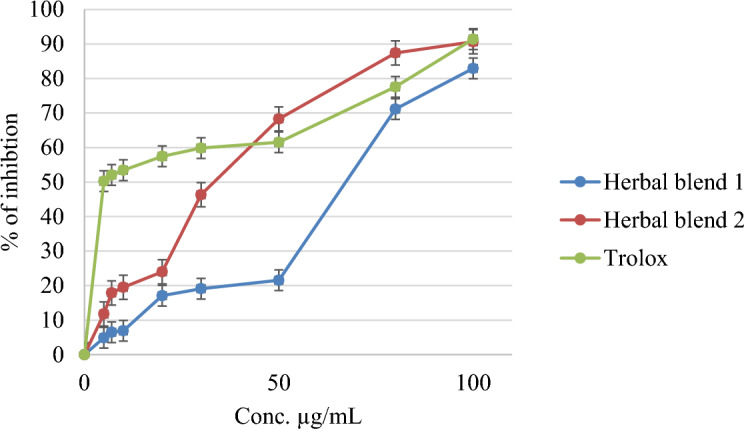
DPPH free radicals scavenging property of two herbal blends and Trolox.

The anti-oxidant activities of the evaluated Herbal blends were evaluated using the DPPH assay, and the results are displayed in Fig. 1. **Herbal Blend 1** exhibited an IC_50_ value of 68.16 ± 2.45 µg/mL, while **Herbal Blend 2** showed an IC_50_ value of 33.97 ± 1.14 µg/mL, compared to a positive control (Trolox) with an IC_50_ of 7.72 ± 1.05 µg/mL, as indicated in Table 2.

**Table 2:** The IC_50_ Values in µg/mL on each biological target of both Herbal blends 1 and 2 in comparison with the positive control.

IC_50_ (µg/mL)			
Biological Targets	Herbal Blend 1	Herbal Blend 2	Control
α-Amylase	468.98 ± 2.44	243.73 ± 1.57	6.42 ± 1.02^a^
Lipase	1466.85 ± 3.54	1358.39 ± 2.04	5.44 ± 1.37^b^
DPPH	68.16 ± 2.45	33.97 ± 1.14	7.72 ± 1.05^c^
Hep3B	148.37 ± 2.45	71.74 ± 1.77	1.21 ± 1.0^d^
MCF-7	127.10 ± 2.08	52.53 ± 0.78	< 1^d^
HeLa	258.06 ± 1.07	164.89 ± 2.01	1.55 ± 1.35^d^
LX-2	83.25 ± 2.45	24.36 ± 0.54	< 1^d^

^a^Acarbose, ^b^Orlistat, ^c^Trolox, and ^d^DOX while *p*-value was < 0.05.

It was found that **Herbal Blend 2** had a more potent antioxidant activity compared to **Herbal Blend 1.** The higher antioxidant activity of Herbal Blend 2 can be attributed to the presence of *C. longa*,* A. sativum*, and *P. nigrum.* In fact,* C. longa* is known to contain curcumin, which has been extensively studied for its potential antioxidant properties^46^. Similarly, *A. sativum* contains sulfur-containing compounds that also possess antioxidant properties. A study published in the Journal of Agricultural and Food Chemistry showed that garlic extracts have high antioxidant activity, as measured by the ORAC (Oxygen Radical Absorbance Capacity) assay^47^. Besides, an investigation established by Nahak and Sahu found that *P. nigrum* fruits exhibited remarkable DPPH free radicals scavenging ability at different concentrations^48^. Moreover, *P. nigrum* enhances the bioavailability of curcumin, thereby increasing the antioxidant potential of Herbal Blend 2. Although Herbal Blend 1 ingredients may also have some antioxidant potential, they may not be as potent as the combination of *C. longa, A. sativum,* and *P. nigrum* found in Herbal Blend 2.

The percentage of inhibition was measured for the Herbal blends at different concentrations, and the results are illustrated in Fig. 1. Herbal Blend 2 showed a significantly higher percentage of inhibition than Herbal Blend 1, and in some concentrations, it even surpassed the control (Trolox). For instance, at a concentration of 50 µg/mL, Herbal Blend 2 exhibited a percentage of inhibition of 68%, while Trolox showed 61%.

Herbal remedies have been used for centuries in traditional medicine for their anticancer and cytotoxic effects. These effects are attributed to the presence of various active compounds in plants, such as triterpene saponins, flavonoids, and phenolic compounds, which have been shown to inhibit cancer cell growth, induce apoptosis, and cause DNA damage^49^.

**Figure 2:**
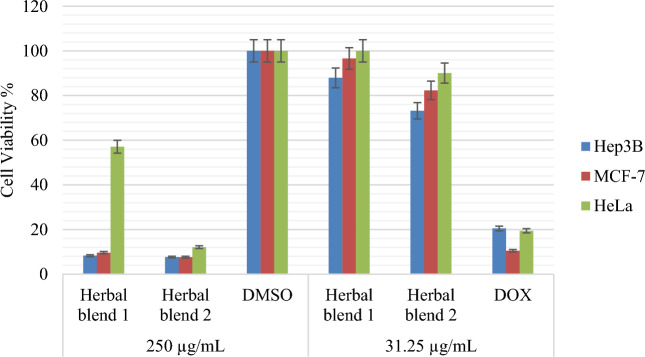
Cytotoxic activity of Two herbal blends, DMSO and DOX on Hep3B, MCF-7, and HeLa cancer cells.

The anti-cancer activities of the tested Herbal blends were evaluated using the MTS assay, and the results showed that **Herbal Blend 2** had potent anticancer activities against liver cancer and breast cancer compared to **Herbal Blend 1,** which also exhibited anticancer activities by inducing apoptosis in liver and breast cancer cells. The percentage of cell viability was measured at different concentrations for the Herbal blends, as shown in Fig. 2**.**

Based on the results of the biological evaluation, it was found that Herbal Blend 2 had a more potent anticancer activity compared to Herbal Blend 1. One possible reason for this difference in activity could be attributed to the presence of *C. longa* and *A. sativum* in Herbal Blend 2. *C. longa* contain curcumin, which has been extensively studied for its anticancer properties, Curcumin, employed in Ayurvedic medicine for its anti-inflammatory properties, has been reported to synergistically inhibit tumor cell growth and induce apoptosis^50^. Similarly, *A. sativum* contains organosulfur compounds including allicin, which have also been shown to possess anticancer properties^51^. *Piper nigrum*, which is present in both Herbal blends, is known to enhance the bioavailability of curcumin, potentially increasing its activity^52^. Additionally, previous studies explored the anticancer activity of *P. nigrum* fruits in both in vitro and in vivo breast cancer models which showed that the treatments with the plant extracts induced intracellular oxidative stress, which was considered the main component responsible for its cytotoxic effects in cancer cells^53^. In contrast, While these ingredients in Herbal Blend 1 may also possess some medicinal properties, they may not have the same level of anticancer activity as *C. longa*. and *A. sativum* found in Herbal Blend 2.

For Hep3B cancer cell lines, the IC_50_ of Herbal Blend 1 was 148.37 ± 2.45 µg/mL, while the IC_50_ of Herbal Blend 2 was 71.74 ± 1.77 µg/mL, compared to a positive control (DOX) with an IC_50_ of 1.21 ± 1.0 µg/mL, as shown in Table 2**.** Similarly, for MCF-7 cells, the IC_50_ of Herbal Blend 1 was 127.10 ± 2.08 µg/mL, while the IC_50_ of Herbal Blend 2 was 52.53 ± 0.78 µg/mL, compared to a positive control (DOX) with an IC_50_ of less than 1, as shown in Table 2. In addition, for HeLa cells, the IC_50_ of Herbal Blend 1 was 258.06 ± 1.07 µg/mL, while the IC_50_ of Herbal Blend 2 was 164.89 µg/mL, compared to a positive control (DOX) with an IC_50_ of 1.55 µg/mL, as presented in Table 2. These results indicate that Herbal Blend 2 has a stronger anti-cancer activity against liver cancer and breast cancer cells compared to Herbal Blend 1.

*C. verum* commonly known as Cinnamon, exhibits anti-cancer, antioxidant, anti-lipid, and anti-fibrotic properties^17,54^ It demonstrates anticancer effects against various cancer cell types, including breast cancer, through apoptosis induction and angiogenesis suppression. This extends to its efficacy against lung and colon cancer cells^55,56^. *C. longa*, or Turmeric, is recognized for its potent anti-cancer properties, effectively inhibiting the growth of various cancer cells such as those in breast, prostate, and colon cancers^57–59^. Additionally, *C. longa* displays significant antioxidant activity, contributing to the prevention of disorders associated with oxidative stress, including cardiovascular diseases and cancer^57,60^. The herb’s anti-lipid properties are notable, demonstrated by its ability to reduce blood glucose and lipid levels in diabetic mice. Furthermore, Curcuma longa showcases anti-fibrotic properties, suggesting potential benefits in preventing and treating fibrotic conditions like liver fibrosis^20,21,61^.

The anti-fibrotic activity of the tested Herbal blends was also evaluated, and it was found that Herbal Blend 2 exhibited more potent anti-fibrotic activity compared to Herbal Blend 1. The IC_50_ values for Herbal Blend 2 and Herbal Blend 1 in the anti-cancer assay were 24.36 ± 0.54 and 83.25 ± 2.45 µg/mL, respectively, while the control drug DOX showed an IC_50_ of < 1, as shown in Table 2. These results suggest that the tested Herbal blends may have potential therapeutic applications not only for fibrotic conditions but also for cancer treatment due to their multi-faceted activities. The findings of this study provide a basis for further research and development of these Herbal blends as potential anti-fibrotic, anti-cancer, and antioxidant agents.

Herbal Blend 2 had a more potent antifibrotic activity compared to Herbal Blend 1. The higher antifibrotic activity of Herbal Blend 2 could be due to the presence of *C. longa* and *A. sativum* in the Herbal blend. Curcumin, present in *C. longa* has been reported to have antifibrotic properties by inhibiting fibrosis-promoting factors^62^. Additionally, *A. sativum* contains sulfur-containing compounds that possess antifibrotic properties^63^. Therefore, the combination of *C. longa* and *A. sativum* in Herbal Blend 2 may be responsible for its higher antifibrotic activity.

When considering all of these activities together, it is evident that Herbal Blend 2 has the potential to provide multiple benefits for individuals with liver cancer. The anti-cancer activity of Herbal Blend 2 against Hep3B cells, combined with its anti-fibrotic activity, may help to prevent the progression of liver fibrosis, a common complication of liver cancer. Furthermore, the antioxidant activity of Herbal Blend 2 may help to protect liver cells from oxidative stress, which is known to contribute to the development and progression of liver cancer. Therefore, the multi-faceted benefits of Herbal Blend 2 make it a promising candidate for further investigation and development as a potential therapeutic agent for liver cancer.

**Figure 3:**
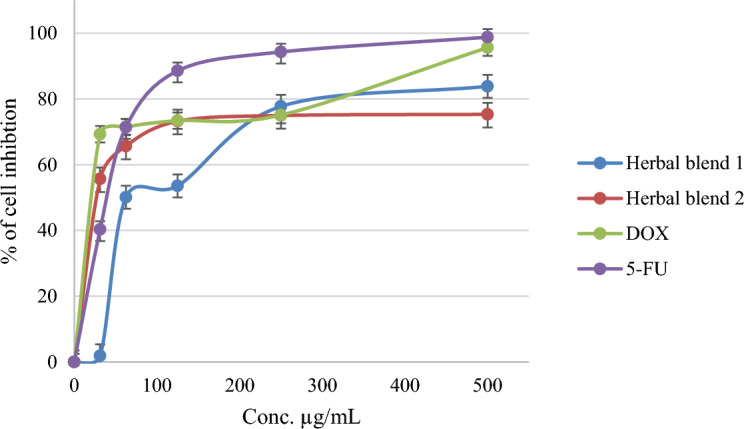
Antifibrotic effect of two herbal blends, 5-FU and DOX on LX-2 cell lines.

Figure 3 displays the percentage of LX-2 cells inhibition at various concentrations for both Herbal Blend 1 and Herbal Blend 2, demonstrating promising results. The inhibition percentages for the Herbal blends were compared with those of 5FU and DOX. At a concentration of 80 µg/mL, Herbal Blend 2 exhibited an inhibition percentage of 68%, while Herbal Blend 1 displayed a percentage of 50%. In contrast, the inhibition percentages of 5-Fu and DOX were 72% and 75%, respectively. Moreover, at a concentration of 250 µg/mL, Herbal Blend 2 demonstrated a percentage of 77%, which was almost equivalent to the percentage of Herbal Blend 1 (77%). However, the LX-2 cells inhibition percentages of 5-Fu and DOX were 95% and 75%, respectively. These results indicate that both Herbal blends have significant inhibitory effects on LX-2 cancer cells and suggest that they could be effective alternatives to traditional chemotherapy drugs.

The anti-diabetic and anti-obesity potential of the tested Herbal blends was evaluated by examining their effects on porcine pancreatic α-amylase and lipase enzymes. The results showed that Herbal Blend 2 exhibited more potent anti-amylase activity compared to Herbal Blend 1, with IC_50_ doses of 243.73 ± 1.57 and 468.98 ± 2.44 µg/mL, respectively. In contrast, the control drug Acarbose displayed an IC_50_ value of 6.42 ± 1.02 µg/mL, indicating that both Herbal blends have moderate anti-amylase effects. However, there was a negligible effect on lipase activity for both Herbal blends, as Herbal Blend 2 and Herbal Blend 1 exhibited IC_50_ values of 1358.39 ± 2.04 and 1466.85 ± 3.54 µg/mL, respectively, whereas the control drug Orlistat had an IC_50_ value of 5.44 µg/mL, as shown in Table 2**.** These findings suggest that the evaluated Herbal blends could be used as natural treatments for diabetes and obesity due to their significant anti-amylase activity with caution because of their potent activities on various cell lines.

Herbal Blend 2 had a more potent anti-amylase activity compared to Herbal Blend 1. The higher anti-amylase activity of Herbal Blend 2 could be due to the presence of *C. verum* and *P. nigrum* in the Herbal blend. Both of these ingredients are known to possess anti-amylase properties. For example, cinnamon extract has been reported to inhibit α-amylase, an enzyme involved in carbohydrate digestion, and reduce the postprandial glucose response^64^. Additionally, *P. nigrum* contains piperine, which has been shown to inhibit α-amylase and reduce starch digestion^65^. Therefore, the combination of *C. verum* and *P. nigrum* in Herbal Blend 2 may be responsible for its higher anti-amylase activity.

Herbal Blend 1 and Herbal Blend 2, revealed that they had a negligible effect on lipase enzyme activity. The reason for this could be due to the absence of specific ingredients in both Herbal blends that are known to have lipase-inhibitory properties. For example, some studies have shown that polyphenols, such as epigallocatechin-3-gallate (EGCG) found in green tea (*C. sinensis*) and curcuminoids found in turmeric (*C. longa* ), can inhibit pancreatic lipase activity^66^. However, the levels of these polyphenols in Herbal Blend 1 and Herbal Blend 2 may not have been sufficient to have a significant impact on lipase enzyme activity.

**Figure 4:**
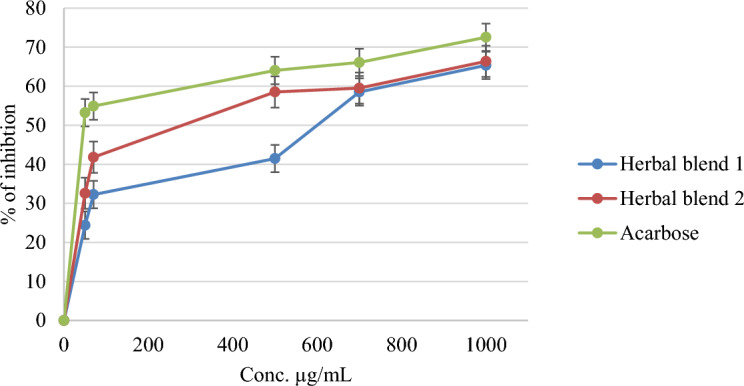
Anti-amylase effect of Two herbal blends and Acarbose.

Figure 4 displays the percentage of inhibition at different concentrations of the tested Herbal blends on α-amylase, and the results were promising for both Herbal blends compared to the control Acarbose. Herbal Blend 2 demonstrated a percentage of inhibition of 59% at a concentration of 510 µg/mL, while Herbal Blend 1 showed 44% inhibition at the same concentration. In comparison, the percentage of inhibition for Acarbose was 64%. At a concentration of 730 µg/mL, both Herbal blends showed similar percentages of inhibition, as Herbal Blend 2 demonstrating a 60% inhibition and Herbal Blend 1 showing 58% of inhibition, while the percentage of inhibition for Acarbose was 67%. These results indicate that both Herbal blends have anti-diabetic activity through their inhibition of α-amylase, and Herbal Blend 2 was found to have slightly more potent activity compared to Herbal Blend 1.

**Figure 5:**
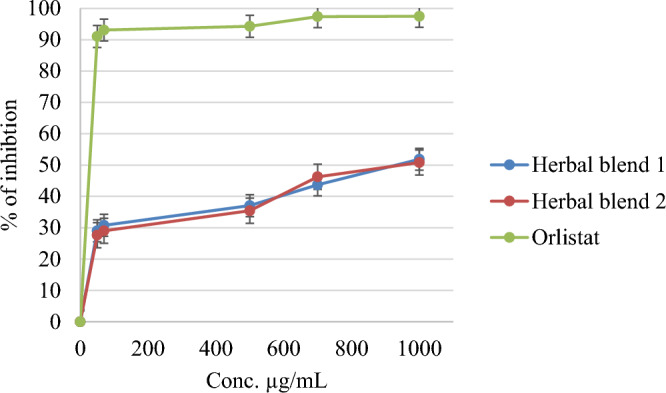
Anti-lipase effect of two Herbal blends and Orlistat.

Figure 5 presents the percentage of inhibition at different concentrations of the two tested Herbal blends against lipase. The results showed negligible effects for both Herbal blends compared to Orlistat. At a concentration of 510 µg/mL, Herbal Blend 2 had almost the same percentage of inhibition as Herbal Blend 1, with Herbal Blend 2 showing a percentage of 35% and Herbal Blend 1 showing 37%, while Orlistat exhibited a percentage of 97%. Similarly, at a concentration of 730 µg/mL, Herbal Blend 2 showed a percentage of 44%, and Herbal Blend 1 showed 42%, while Orlistat exhibited a percentage of 99%. These results suggest that the two tested Herbal blends have a negligible effect on lipase activity compared to Orlistat.

Further standardization of the active components in the investigated herbal blends, along with in vivo and preclinical research, is necessary to validate the findings of our study and develop appropriate pharmaceutical dosages from these traditional blends to enhance community healthcare.

## Conclusion

In conclusion, our study provides valuable insight into the potential use of these plant Herbal blends for their anti-obesity and other health benefits. While we found that these plants have a moderate effect as anti-obesity agents, our research demonstrated potent anticancer, antioxidant, and antifibrotic effects. This presents a unique challenge, as many anticancer plants are known to have cytotoxic effects on the body. Therefore, we strongly recommend that any use of these plant Herbal blends and plants, in general, should be under the supervision of a qualified plant specialist and the Palestinian health authorities. It is essential to exercise caution when using natural remedies, and we urge individuals to seek medical advice before incorporating them into their health regimens.

## Data Availability

The data sets used and/or analyzed during the current study are available from the corresponding author upon reasonable request.
